# Platelet-Expressed Synaptophysin (pSyn) as Novel Biomarker in Neuroendocrine Malignancies

**DOI:** 10.3390/cancers13102286

**Published:** 2021-05-11

**Authors:** Martina Hinterleitner, Bence Sipos, Verena Wagner, Julia M. Grottenthaler, Ulrich M. Lauer, Lars Zender, Clemens Hinterleitner

**Affiliations:** 1 Department of Medical Oncology and Pneumology (Internal Medicine VIII), University Hospital Tuebingen, 72076 Tuebingen, Germany; Martina.Hinterleitner@med.uni-tuebingen.de (M.H.); Bence.Sipos@med.uni-tuebingen.de (B.S.); Ulrich.Lauer@med.uni-tuebingen.de (U.M.L.); Lars.Zender@med.uni-tuebingen.de (L.Z.); 2 Cluster of Excellence iFIT (EXC 2180) “Image-Guided and Functionally Instructed Tumor Therapies”, University of Tuebingen, 72076 Tuebingen, Germany; 3 Institute of Clinical Sciences, Faculty of Medicine, Imperial College London, London W12 0NN, UK; V.Wagner@lms.mrc.ac.uk; 4 MRC London Institute of Medical Sciences (LMS), London W12 0NN, UK; 5 Department of Gastroenterology, Gastrointestinal Oncology, Hepatology, Infectiology, and Geriatrics (Internal Medicine I), University Hospital Tuebingen, 72076 Tuebingen, Germany; Julia.Grottenthaler@med.uni-tuebingen.de; 6 German Cancer Research Consortium (DKTK), Partner Site Tuebingen, German Cancer Research Center (DKFZ), 69120 Heidelberg, Germany

**Keywords:** neuroendocrine malignancies, biomarker, synaptophysin, platelets

## Abstract

**Simple Summary:**

This study describes the expression of synaptophysin on platelet surfaces of neuroendocrine neoplasms (NENs). Compared to healthy donors, platelet-expressed synaptophysin was shown to be significantly upregulated in NENs patients. Platelet-expressed synaptophysin was significantly correlated with tumor proliferation and metastasis, demonstrating the involvement of platelets in tumor biology. Expression of synaptophysin on platelet surfaces was finally shown to predict progression-free survival in NEN. This study conceptually explored platelet-expressed synaptophysin as a novel biomarker in NEN.

**Abstract:**

Neuroendocrine neoplasms (NENs) encompass a heterogeneous group of tumors. Whereas low-grade neuroendocrine tumors (NETs) are histologically well-differentiated, highly aggressive neuroendocrine carcinomas (NECs) are characterized by a high proliferation rate and a worse clinical outcome. Since most NEN patients need monitoring of tumor progress and response to treatment for a long period of time, especially in metastatic disease, reliable, dynamic, and easy-to-assess biomarkers are needed. In this prospective study, we identified platelet-expressed synaptophysin (pSyn) as a novel biomarker in NENs. The level of pSyn in NENs was significantly upregulated compared to healthy donors. pSyn was positively correlated with higher tumor stages, the occurrence of metastasis, histological grading, and higher tumor proliferation (Ki67). Most importantly, high pSyn expression in our NEN cohort was shown to predict shorter progression-free survival (PFS). In conclusion, our data highlight the potential of pSyn as a novel biomarker in NENs reflecting tumor stages, grading, and prognosis.

## 1. Introduction

Neuroendocrine neoplasms (NENs) represent a rare heterogenic group of tumors. They can develop in multiple organs showing the highest frequency in the lung, small bowel, and pancreas [1]. NENs originate from neuroendocrine cells, which are involved in hormone homeostasis via the release of bioactive substances like insulin, somatostatin, gastrin or serotonin [1,2]. Regardless of their organ of origin, NENs can be divided into two major groups based on differentiation and proliferation rate (Ki67-rate): low-grade neuroendocrine tumors (NETs) and highly-aggressive neuroendocrine carcinomas (NECs) [1]. Low-grade NETs are commonly associated with a mild clinical course, even in a metastatic disease [3]. In contrast, high-grade NECs are characterized by a high proliferative capacity and a worse clinical outcome [2]. High-grade NECs can either develop primarily, or develop as a component of mixed non-neuroendocrine-neuroendocrine carcinomas (MiNEN). In addition, treatment-associated dedifferentiation of NETs to higher proliferating NET (NET G3) and maybe even into NEC can be observed [4,5].

Chromogranin A (CgA) is a widely used serum biomarker that has a limited sensitivity of 66–73% [6,7]. CgA serum levels are elevated in several diseases that are unrelated to NENs including chronic gastritis, renal or hepatic failure, inflammatory bowel disease, and pancreatitis. In addition, several medications affect serum CgA levels, e.g., proton pump inhibitors (PPIs), somatostatin analogues and steroids. Finally, there are NEN, which express only low levels of tissue CgA like rectal NET and up to 45% of NEC. The second most utilized NEN serum marker, neuron-specific enolase (NSE), exhibits a sensitivity of approximately 30% [8]. DOTATATE/DOTATOC- and/or FDG-PET scans are well established diagnostic tools in NETs and NECs, however, the availability is restricted in several countries. Thus, further reliable and affordable biomarkers of NEN are undoubtedly needed.

Since the expression of synaptophysin as an integral membrane glycoprotein in presynaptic vesicles is highly specific for neuroendocrine cells, it provides all requirements for a promising static, tissue-based biomarker in NENs [9]. Furthermore, synaptophysin is the most reliable tissue marker and indispensable for the histological diagnosis of NEN [10]. Of note, the implementation of synaptophysin as a dynamic liquid biomarker, via its expression on circulating tumor cells (CTCs) for example, remains challenging [11]. Moreover, only very limited data exist on soluble synaptophysin so far [12]. Since platelets are directly related to the development, progression, and metastasis of tumors, they represent a promising target for both diagnostic and interventional approaches [13,14,15]. The pathophysiological role of platelets in the tumor microenvironment (TME) encompasses the supply of growth factors, the induction of epithelial-to-mesenchymal transition (EMT), the mediation of endothelial adhesion and transmigration of tumor cells, and the contribution to immune evasion processes [16,17,18]. Of note, in addition to tumor-specific mRNA, which can be detected in tumor-educated platelets (TEP), protein expression of different TNF family members on platelets shows potential as a promising tumor biomarker [19,20,21,22].

In this prospective study, we evaluated the expression of synaptophysin on platelets of patients with neuroendocrine malignancies. Platelet-expressed synaptophysin (pSyn) was found to be significantly upregulated during tumor progression. Remarkably, pSyn was shown to be of prognostic and predictive relevance in NEN and might therefore be suitable as a novel platelet-based liquid biomarker.

## 2. Materials and Methods

### 2.1. Reagents

Paraformaldehyde was purchased from Affymetrix (Santa Clara, CA, USA). Anti-human synaptophysin antibody (APC labeled) and the respective isotype control were obtained from Miltenyi Biotec (Bergisch Gladbach, Germany). CD41a-VioBlue and CD62P-FITC were from Miltenyi Biotec (Bergisch Gladbach, Germany) and BD Pharmingen (San Diego, CA, USA). Citrate buffer contained 128 mmol/L NaCl, 11 mmol/L glucose, 7.5 mmol/L Na2HPO4, 4.8 mmol/L sodium citrate, 4.3 mmol/L NaH2PO4, 2.4 citric acid, 0.35% bovine serum albumin, and 50 ng/mL prostaglandin E1.

### 2.2. Patient Characteristics

In 2020, blood samples from 54 consecutive patients with histopathologically confirmed neuroendocrine malignancies, treated at the Department of Medical Oncology and Pneumology (University Hospital Tuebingen, Germany), were included in our screening cohort (SC). Written informed consent in accordance with the Helsinki protocol was given in all cases. The patient characteristics in detail are given in Table 1 and Table S1. All gastroenteropancreatic NET patients in our study were classified according to the WHO classification of 2019 [23]. Two lung NET patients were classified according the WHO 2015 classification [24], (patient I: typical carcinoid, T1, N0, M0, 2 mitosis/10 HPF, no necrosis; patient II: atypical carcinoid, T2, N2, M1, 3 mitosis/4 HPF, necrosis present). To improve comparability in our heterogeneous NEN cohort, we also used Ki67 in these patients. In our proof of principle cohort (PoP), three patients with additional tumor entities (chronic lymphatic leukemia, chronic myelomonocytic leukemia, and breast cancer) were subsequently excluded from further analysis. 

### 2.3. Preparation of Platelets

Platelets were obtained from 20 healthy donors (HD) (not taking any medication for at least 10 days) and 54 NEN patients after informed written consent as previously described [17].

### 2.4. Immunohistochemistry

Paraffin-embedded tumor sections were incubated with primary antibodies against synaptophysin (clone SP11). The specific antibody binding was visualized using the Roche Ventana Ultra Immunostainer (DAB, Roche, Basel, Switzerland ). Hematoxylin-eosin was used as counterstaining. For immunofluorescence analysis, platelets of NEN patients and healthy donors were fixed in 4% PFA in PBS (10 min at 4 °C). Platelets were then blocked using a blocking solution containing 5% BSA, 0.2% Triton X-100, 0.1% Tween for 60 min. As primary antibody, we used anti-synaptophysin (1:200, Invitrogen, Carlsbad, CA, USA) and anti-CD41 (1:500, ThermoFisher, St. Louis, MO, USA); as secondary antibodies, Alexa-Fluor 594 labelled anti-rabbit (1:1000, Invitrogen, Carlsbad, CA, USA) and Fluor 488 labelled anti-mouse (1:1000, Invitrogen) were used. Slides were mounted in fluorescent mounting medium. Pictures were acquired using an Olympus BX63 microscope and a DP80 camera (Olympus, Shinjuku, Japan).

### 2.5. Flow Cytometry

Flow cytometry was performed using fluorescence-conjugates at saturating concentrations. Analysis was performed using a FACS Canto (BD Biosciences, Heidelberg, Germany). Platelets were selected as subcellular CD41a+ and CD62P− (resting) or CD62P+ (activated) cells. Percent positive platelets were calculated as follows: “percent surface expression obtained with specific antibody” minus “percent surface expression obtained with isotype control”.

### 2.6. Statistics

For continuous variables student’s *t*-test, Mann-Whitney U test or one-way ANOVA were used. For categorical data, we used chi-squared test or Fisher’s exact test. Correlation of pSyn and hematological parameters, Ki67 expression, NSE, and CgA were analyzed using simple linear regression analysis. The predictive value of pSyn was evaluated by examining the area under the receiver operating characteristic (ROC) curve with a confidence interval of 95%. The cut-off value to determine pSyn high (pSynhi) and pSyn low (pSynlo) was determined using Youden’s J statistic. Progression-free survival (PFS), including the median, was calculated using the Kaplan-Meier method. Hazard ratios (HRs) were determined using Cox regression analysis. A multivariate analysis to predict pSyn was performed using a linear regression analysis. All statistical tests were considered significant when *p* was below 0.05.

## 3. Results

### 3.1. Expression of Synaptophysin in Tumor Tissue and Platelets Derived from Patients with Neuroendocrine Neoplasm (NEN)

**Figure 1:**
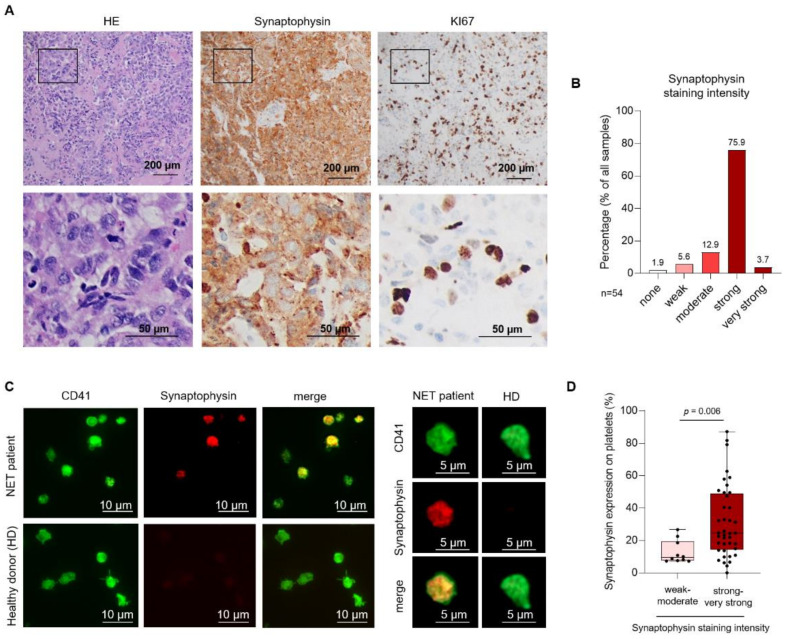
Expression of Synaptophysin in tumor tissue and platelets derived from neuroendocrine neoplasm (NEN) patients. (**A**) Representative HE, Syn, and Ki67 staining in a NET G2 patient. The proliferation index (Ki67) was calculated as 10%. Images taken at 10× magnification. (**B**) Relative Syn staining intensity in the NEN cohort of 54 patients. (**C**) Immunofluorescence analysis of platelet-expressed Syn (labeled in red) in a NET G3 patient and a HD. Platelets were identified via CD41 (labeled in green). Images taken at 60× magnification. (**D**) Correlation of synaptophysin expression on platelets and synaptophysin staining intensity in tumor tissue.

Synaptophysin (Syn) mediates several roles in synaptic functions including synaptic formation, exo- or endocytosis of neuroendocrine cells [25]. Beyond that, synaptophysin serves as a well-established marker protein with a high sensitivity and specificity for NENs [9]. To validate synaptophysin as a tissue marker of NENs, we first stained neuroendocrine tumors of 54 patients. In our cohort of 54 NENs, synaptophysin was expressed in 98.15% of all cases (53 patients) (Figure 1A). In 94.4% of all samples (51 patients), synaptophysin staining intensity was moderate, strong, or very strong (Figure 1B). Weak staining was seen in three patient samples. It has been described that tumor cells interact with platelets via an active transfer of tumor-specific molecules to platelets [26]. We therefore hypothesized that platelets, derived from patients with neuroendocrine malignancies, might express distinct levels of tumor-specific surface proteins like synaptophysin. In a first step, we performed immunofluorescence analysis of platelets derived from patients with histologically confirmed NEN and healthy donors (HD). Platelets were identified by the platelet integrin IIb (CD41). In several platelets, derived from NEN patients, we observed a relevant synaptophysin expression. In contrast, platelet samples from HD showed no relevant staining for synaptophysin (Figure 1C). Remarkably, compared to patients displaying a weak to moderate staining intensity in the tumor, patients showing a strong or very strong synaptophysin staining displayed higher pSyn level (Figure 1D). 

### 3.2. Characterization of Platelet-Expressed Synaptophysin (pSyn) in NEN Patients and HD

**Figure 2:**
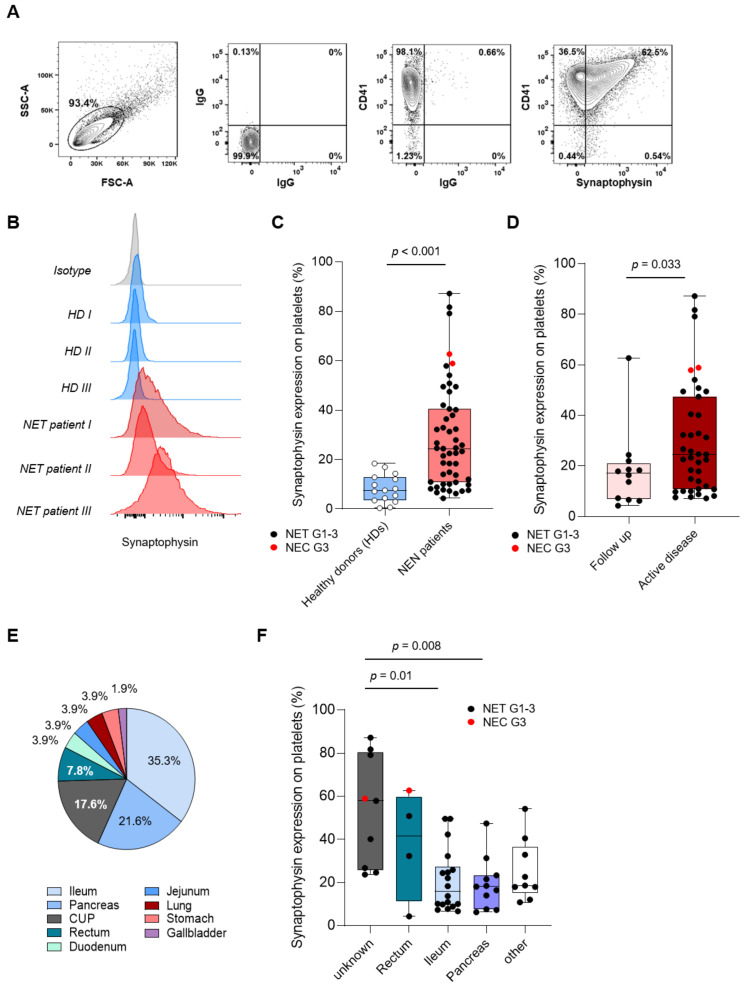
Characterization of pSyn in NEN patients and HD. (**A**) Gating strategy used to analyze pSyn expression on platelets ex vivo using flow cytometry. (**B**) Representative results for pSyn expression obtained from patients (*n* = 3) and HDs (*n* = 3) are shown. (**C**) pSyn expression of 20 HD and 51 NEN patients in the PoP cohort is shown. (**D**) pSyn expression levels in NEN patients receiving follow-up care and active disease. (**E**) Primary tumor origins of the NEN PoP cohort (*n* = 51). (**F**) pSyn expression in accordance to primary tumor origin.

Since our immunofluorescence analysis indicates that synaptophysin seems to be differentially expressed on platelets derived from NEN patients and HD, we further validated these findings via flow cytometry (Figure 2A). In line with our previous findings, we observed a significant upregulation of synaptophysin on platelets derived from NEN patients in the PoP cohort (NEN patients (*n* = 51): 22.95%, 95%CI: 3.82–79.75% vs. HD (*n* = 20): 5.85%, 95%CI: 0–18.33%, *p* < 0.001, Figure 2B,C). In contrast to HD, NEN patients displayed a wide range of inter-individual variability. Of note, expression levels of pSyn in our small cohort of NEN were not associated with age, sex, comorbidities, or medications affecting platelet function (Figure S1).

Accordingly, we analyzed platelet-expressed synaptophysin (pSyn) level in the subgroup of patients displaying an active tumor disease and compared them with patients receiving follow-up care. Interestingly, compared to follow-up patients, NEN patients with an active tumor disease showed significantly increased level of pSyn (Follow-up: 17.9%, 95%CI: 4.3–62.7% vs. active disease: 24.7%, 95%CI: 7.6–82%, *p* = 0.04, Figure 2D). However, pSyn level in patients receiving follow-up care were still significantly increased compared to HD (*p* = 0.001, Figure S1).

Since neuroendocrine malignancies arise from multiple origins, we further investigated whether different tumor origins of ours correlate with different expression levels of pSyn on the platelets. The distribution of the tumor origins in our NEN cohort is shown in Figure 2E. Patients with pancreatic NET (18.1%, 95%CI: 6.2–47.4%) and midgut NET (16%, 95%CI: 6.6–49.5%) showed the lowest pSyn level. Of note, patients with unknown primary tumor origin displayed the highest pSyn level in the entire cohort (57.9%, 95%CI: 23.7–87.2%) (Figure 2F).

### 3.3. Association of pSyn with Hematological Parameters

**Figure 3:**
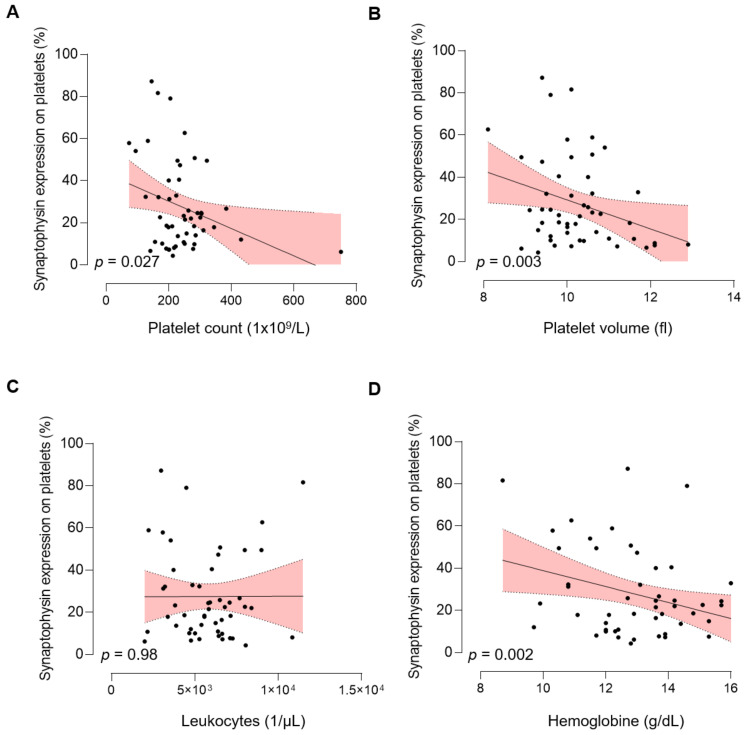
Association of pSyn with hematological parameters. (**A**) Correlation of pSyn expression and platelet count, platelet volume (**B**), leukocyte count (**C**), and hemoglobin concentration (**D**). Line indicates linear regression, gray area 95% CI interval.

Our observation that patients with active tumor disease display significantly increased levels of pSyn (Figure 2D) might indicate a possible relationship between pSyn and TME and/or tumor progression in NEN. Since it has been described that “tumor-educated” platelets can differ in platelet count and volume, caused by a manifold of mechanisms [27], we investigated the association of pSyn with different hematological parameters. Notably, the fraction of synaptophysin positive platelets was negatively correlated with platelet count (*p* = 0.027, Figure 3A), platelet volume (*p* = 0.003, Figure 3B), and higher platelet activation (CD62P) level (*p* < 0.001, Figure S2). Whereas no correlation between pSyn and leukocyte count (*p* = 0.98, Figure 3C) was observed, pSyn showed a negative correlation with hemoglobin levels (*p* = 0.002, Figure 3D). In summary, pSyn level seems to be directly related to detectable changes in the peripheral blood count.

### 3.4. Correlation of pSyn with Different Tumor Characteristics in NEN

**Figure 4:**
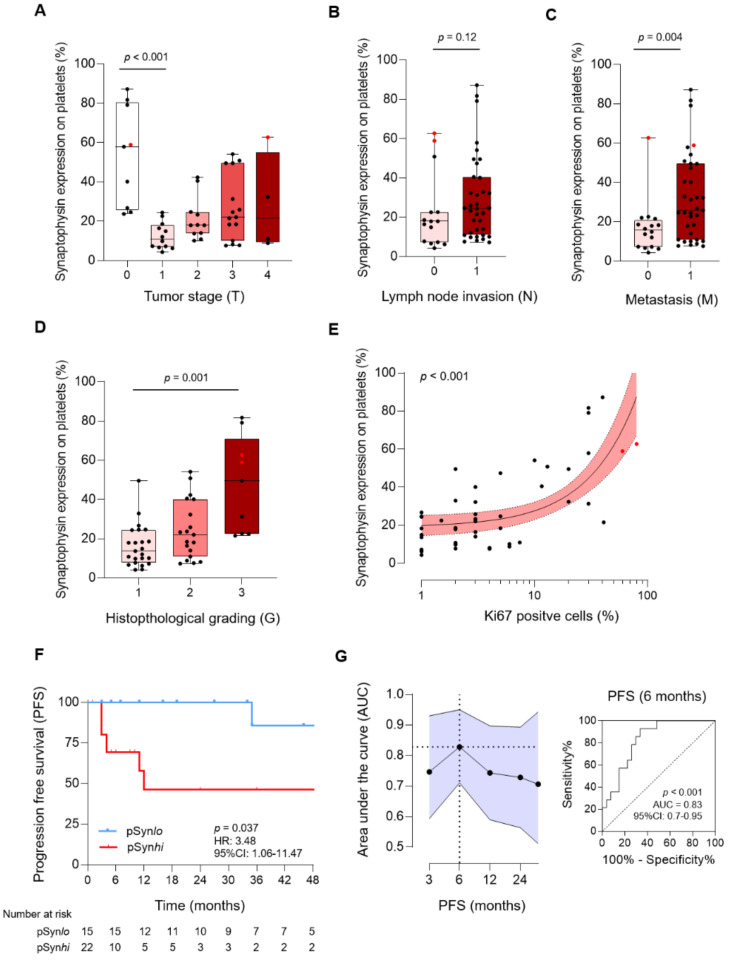
Associations of pSyn with tumor characteristics in NEN. (**A**) Expression of pSyn according to tumor stages (T), (**B**) lymph node invasion (N), and metastasis (M) (**C**). (**D**) Correlation of pSyn and histological grading (G). (**E**) Correlation of pSyn level and tumor proliferation (% Ki67 positive tumor cells). Line indicates linear regression, gray area 95% CI interval. (**F**) Kaplan-Meier analysis for PFS in pSyn high (pSynhi) and pSyn low (pSynlo) expressing patients. (**G**) The predictive value of pSyn expression for PFS, analyzed via ROC-analysis, is shown (black dots represent NET G1-3, red dots represent NEC G3). AUC = Area under the curve; HR = Hazard ratio; CI = Confidence interval; PFS = Progression free survival.

To further investigate our observation that pSyn correlates with an active tumor disease in NEN, we investigated the relationship between pSyn and several tumor characteristics. pSyn was shown to be regulated among different tumor stages (T). Compared to patients with low tumor stages (T1), patients presenting with higher tumor stages (T3) showed significantly increased pSyn levels (*p* = 0.04, Figure 4A). Remarkably, in the subgroup of patients with no detectable primary tumor site (T0), we observed the highest level of pSyn (40.1%, 95%CI: 22.5–81.7%, *p* < 0.001, Figure 4A). In line with these observations, patients with lymph node invasion (N0 vs. N1) tended to have higher pSyn levels (*p* = 0.12, Figure 4B). Most strikingly, patients with metastatic disease displayed significantly increased pSyn levels (*p* = 0.004, Figure 4C).

In accordance with these findings, we observed a positive correlation of pSyn and histological grading (G). Compared to patients with histological grade 1 (G1) tumors, patients with moderately (G2) or poorly differentiated (G3) tumors showed increased levels of pSyn (*p* = 0.001, Figure 4D). As a consequence, pSyn was also found to be positively correlated with tumor cell proliferation (Ki67 level), (*p* < 0.001, Figure 4E). In the next step, we investigated whether pSyn is able to predict progression-free survival (PFS) in patients showing an active disease (*n* = 38) and thus shows potential as a novel predictive biomarker in NEN. In order to establish a cut-off value to define pSyn high (pSynhi) and pSyn low (pSynlo) levels, in NEN we used Youden’s J statistic. Notably, compared to pSynlo patients, individuals in the pSynhi group showed a significantly shortened PFS (Median PFS: 12 months in pSynhi vs. 61 months in pSynlo, median follow-up: 19.7 months, *p* = 0.037, Figure 4F). The HR in our cohort was 3.48 (95%CI of ratio: 1.1–11.5). Finally, we analyzed the validity of pSyn to predict the PFS as a function of time. As shown in Figure 4G, a maximum accuracy of pSyn to predict PFS was 6 months (AUC: 0.83, 95%CI: 07–0.95, *p* < 0.001). In conclusion, determination of pSyn expression was shown to be highly prognostic in NEN. Finally, we reevaluated our follow-up patients 6 months after study inclusion. Of note, three patients, all presenting significantly increased pSyn level (*p* = 0.03), showed a relapse within the first 3 months. However, according to the PFS, this trend was not statistically significant (*p* = 0.14, Figure S3).

### 3.5. Correlation of pSyn with Routine Biomarkers in NEN

**Figure 5:**
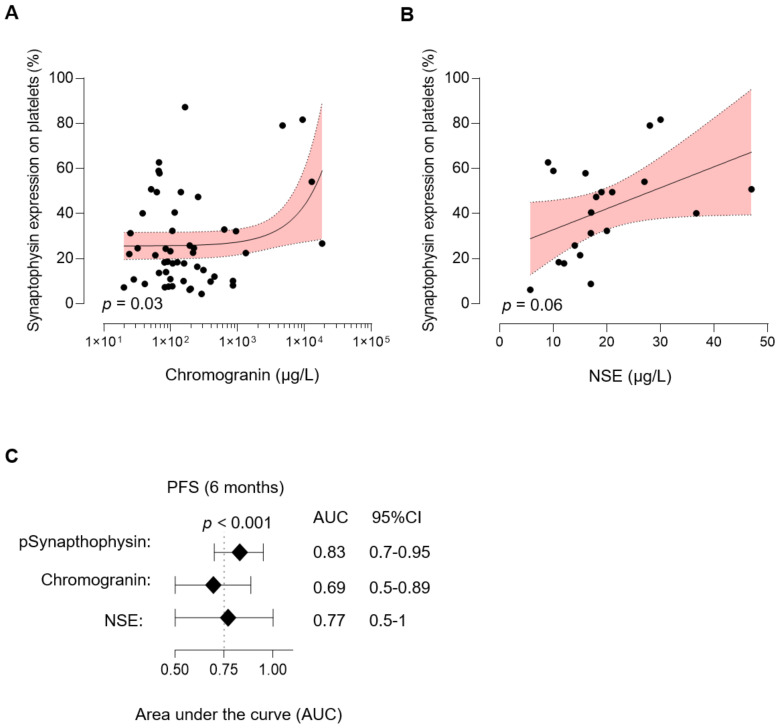
Association of pSyn with routine biomarkers in NEN. (**A**) Correlation of pSyn and CgA. Line indicates linear regression, gray area 95% CI interval. (**B**) Correlation of pSyn and NSE. Line indicates linear regression, gray area 95% CI interval. (**C**) Predictive value of pSyn, CgA, and NSE for PFS (6 months).

Finally, we comparatively analyzed pSyn with NSE and CgA, two markers routinely determined in NET. Interestingly, we observed a positive correlation of pSyn and CgA levels (*p* = 0.03, Figure 5A) and pSyn tended to be positively correlated with NSE; however, this trend was not statistically significant (*p* = 0.06, Figure 5B). Remarkably, the accuracy of pSyn (AUC 0.83, 95%CI: 0.7–0.95, *p* < 0.001) to predict PFS was superior compared to CgA (AUC 0.69, 95%CI: 0.5–0.89, *p* = 0.074) and NSE (AUC 0.77, 95%CI: 0.5–1, *p* = 0.041) (Figure 5C). In conclusion, these data highlight the potential of pSyn as a novel predictive liquid biomarker in NEN.

## 4. Discussion

Neuroendocrine malignancies represent as a rare, heterogeneous group of neoplasms [1]. Irrespective of the primary tumor site, neuroendocrine neoplasms (NENs) share a neuroendocrine differentiation reflected via the expression of CgA, Syn, and neural cell adhesion molecule (NCAM = CD56) [28,29]. Neuroendocrine malignancies vary significantly in their biological behavior. Since low-grade neuroendocrine tumors, associated with a favorable clinical prognosis, can evolve into high-grade NET with worse clinical outcome, dynamic biomarkers are mandatory for prompt diagnosis and optimal treatment [2,4]. However, choosing the optimal diagnostic approach to identify NEN patients with a high risk for tumor progression is challenging. Even if consecutive tumor biopsies and determination of tumor proliferation rate (Ki67) are still the method of choice [1,30], there is a significant peri-interventional risk for patients.

As a result, several non-invasive biomarkers in NEN have been established so far [31]. PET CT scans with ^18^F-FDG as a surrogate marker for tumor dynamics can be used in patients with high grade NET and NEC, as the metabolic activity of these tumors is high [32]. In patients with low-grade NET, PET CT scans with somatostatin analogs such as 68Ga-DOTATATE are the method of choice [33]. In NET patients showing an intermediate proliferation rate, the selection of the optimal imaging modality can be challenging. Serum CgA can easily be determined as a dynamic, liquid biomarker in NET [34]. However, the predictive power of CgA in NEN is limited by its susceptibility to interference by drugs (e.g., proton pump inhibitors) [35], impaired kidney function [36], heart diseases [37], or rheumatoid diseases [38].

In this prospective study, we show, to our knowledge, for the first time, that synaptophysin, an integral membrane glycoprotein in presynaptic vesicles, which is typically expressed in NEN, can be detected on platelets. Whereas the role of synaptophysin in platelets is completely unknown so far, in neurons, synaptophysin bind to the vesicle protein synaptobrevin, thus modulating the formation and fusion of synaptic vesicles with the plasma membrane [39]. The binding partner synaptobrevin, as a vesicle associated membrane protein (VAMP), is known to be expressed in human platelets and has been discussed to modulate platelet exocytosis [8]. Even if further work is needed to reveal the role of synaptophysin as a binding partner of synaptobrevin in tumor-educated platelets, an involvement in platelet exocytosis might be speculated.

Even if platelet-expressed synaptophysin (pSyn) was not shown to be correlated with age, sex, comorbidities, and medication, pSyn was correlated with the staining intensity in the tumor tissue. This might indicate a direct link of synaptophysin expression in the tumor and pSyn expression on platelets. In line with this, we observed significantly increased pSyn level in NEN patients compared to healthy donors (HD). The finding that pSyn levels were higher in patients with an active tumor disease and in patients with an unknown primary tumor site, which is frequently associated with a higher tumor stage and the occurrence of metastases, might reflect a relevant link between NEN cells and platelets in the TME. This assumption is further supported by findings of Xu et al. [40] who demonstrated that tumor-infiltrating platelets in tumor sections of pancreatic neuroendocrine tumors (pNETs) are predictive for both overall survival (OS) and recurrence-free survival [40]. In addition, the platelet size has been described to have a diagnostic value to distinguish pNET from pancreatic adenocarcinomas [41]. In order to address whether pSyn levels might be associated with different platelet parameters, we correlated pSyn with platelet count and size in our cohort. Our observation that pSyn negatively correlates with both platelet count and size might indicate a close relationship between pSyn, platelet homeostasis, and NEN. The negative correlation of pSyn with hemoglobin level in our context might reflect patients underwent cytotoxic tumor treatment or tumor anemia.

Next, we addressed the question of whether pSyn might be associated with tumor characteristics in NEN. The expression of pSyn was upregulated in advanced diseases, which is reflected by tumor stages (T), lymph node invasion (N), and the occurrence of metastasis (M). In line with our observation that patients with an unknown primary tumor origin showed higher expression of pSyn, patients with no detectable primary tumor size (T0) expressed the highest levels of pSyn. The assumption that expression of pSyn identifies a subgroup of NEN patients displaying an aggressive tumor phenotype is further supported by the finding that pSyn was positively correlated with histological grading and tumor proliferation (Ki67). Most importantly, in our small cohort of NEN patients with an active disease, pSyn was able to predict PFS. Our observation that disease recurrence in the follow-up cohort was also correlated with elevated pSyn levels further highlights the potential of pSyn as a promising marker in NEN.

Nevertheless, our study has some limitations. Since several tumor characteristics are largely dependent on each other, it is difficult to assign pSyn to a pathophysiological context based on our data. In order to evaluate the role of pSyn in NEN more independently, we additionally calculated a multivariable model to predict pSyn (*p* < 0.001, R^2^ = 0.629). In this model only, grading (*p* = 0.032) and metastasis (*p* = 0.005) added statistical significance to our prediction model. However, the small number of patient samples used in our study has to be taken into account. Even if further work is definitively required to understand the relationship between pSyn and tumor progression in NEN, one might speculate that higher tumor burden promotes platelet tumor cell contact in vivo. As a result, certain processes like trogocytosis, i.e., transfer of membrane molecules between tumor cells and platelets, might contribute to explain the observed phenomena [42]. Supporting this hypothesis, we observed a positive correlation of activated platelets and pSyn levels in our cohort. To finally justify pSyn as a dynamic and predictive biomarker in NEN, and to analyze the relationship of pSyn and progression from NET G1/G2 to NET G3 or a presumed high-grade progression from NET G3 to NEC, which is still controversial, sequential pSyn analysis in a larger prospective NEN cohort is additionally needed. Of note, since our study only contains two NEC patients, this study cannot provide any evidence on the relationship of pSyn and tumor progression in NEC.

Several new biomarkers have been investigated in NEN. Beside microRNA (miRNA), in which measurement is challenging and not scandalized [43], circulating tumor cells (CTC) have been evaluated as liquid biopsy in NEN [44]. However, CTCs in NEN were shown not to be sensitive or specific for the detection and diagnosis of NEN. Furthermore, CTCs were considered not to correlate with tumor burden and clinical outcome [44]. The NETest represents a dynamic, blood-based tool for characterizing circulating real-time genetic multianalyte information in NEN [45]. Even if the NETest shows promising results in predicting tumor progression and response to treatment, the multistep protocol used to provide a multianalyte gene expression is still complex.

Due to the fact that pSyn can be easily determined through liquid biopsies, it shows exceptional potential as a new non-invasive liquid biomarker in NEN. It is particularly noteworthy that pSyn was superior to the prediction of PFS compared to CgA and NSE.

In addition to recent data on the predictive role of tumor-specific mRNA signatures in platelets, we show in this study that also tumor-associated proteins like Syn can be detected on platelet surfaces using flow cytometry [13]. This approach provides a new cost- and time-effective diagnostic tool with remarkable potential in cancer. Finally, the observation that pSyn in NEN mimics tumor burden as well as tumor proliferation highlights the potential of tumor-educated platelets as a dynamic liquid biomarker in cancer. 

## 5. Conclusions

In this study we provide evidence that patients with neuroendocrine malignancies display increased levels of synaptophysin on their platelets. As pSyn was significantly associated with tumor progression and metastasis, platelet-expressed synaptophysin shows great potential as a novel, dynamic biomarker in neuroendocrine malignancies.

## Figures and Tables

**Table 1:** Patient characteristics of the NEN screening cohort (SC).

Patient Characteristics	Total (*n* = 54)
Gender	
female sex, *n* (%)	26 (48.2)
Age	
Age in years, mean–yr. ±SD (range)	61 ± 14 (21 to 91)
TNM classification, *n* (%)	
Tumor	
T0	9 (16.7)
T1	13 (24.1)
T2	12 (22.2)
T3	15 (27.8)
T4	5 (3.7)
Node	
N0	15 (27.8)
N1	37 (68.5)
N2	2 (3.7)
Metastasis	
M0	17 (31.5)
M1	37 (68.5)
Histological grading, *n* (%)	
NET G1	23 (42.6)
NET G2	22 (40.7)
NET G3	7 (12.9)
NEC G3	2 (3.7)
Histological characteristics	
Synaptophysin positive, *n* (%)	53 (98.1)
CD56 positive, *n* (%)	24 (44.4)
Current Treatment, *n* (%)	
Somatostatin analogues	26 (48.1)
Targeted therapy (Everolimus)	1 (1.9)
Chemotherapy	9 (16.7)

*n* = number, T = tumor, N = lymph node, M = metastasis, % = percentage, yr. = years, SD = standard deviation, G = grading.

## Data Availability

The data that support the findings of this study are available from the corresponding author upon reasonable request.

## Supplementary Materials

The following are available online at https://www.mdpi.com/article/10.3390/cancers13102286/s1, Figure S1. Correlation of pSyn expression and clinical parameters in NEN. Figure S2. Association of pSyn and platelet activation. Figure S3. Expression of pSyn in NEN patients showing a disease relapse. Table S1. Clinical characteristics of the NEN screening cohort (SC).
