# Supplementary material for: Platelet-Expressed Synaptophysin (pSyn) as Novel Biomarker in Neuroendocrine Malignancies
Source: Cancers (Basel). 2021 May 11;13(10):2286. doi: 10.3390/cancers13102286 (PMC8150833; doi:10.3390/cancers13102286)
Supplement: Supplementary file 1 [file cancers-13-02286-s001.zip › cancers-1187057-supplementary.pdf]

cancers

MDPI

Supplementary Materials

Platelet-Expressed Synaptophysin (pSyn) as Novel Biomarker in Neuroendocrine Malignancies

Martina Hinterleitner, Bence Sipos, Verena Wagner, Julia M Grottenthaler, Ulrich M Lauer, Lars Zender and Clemens Hinterleitner

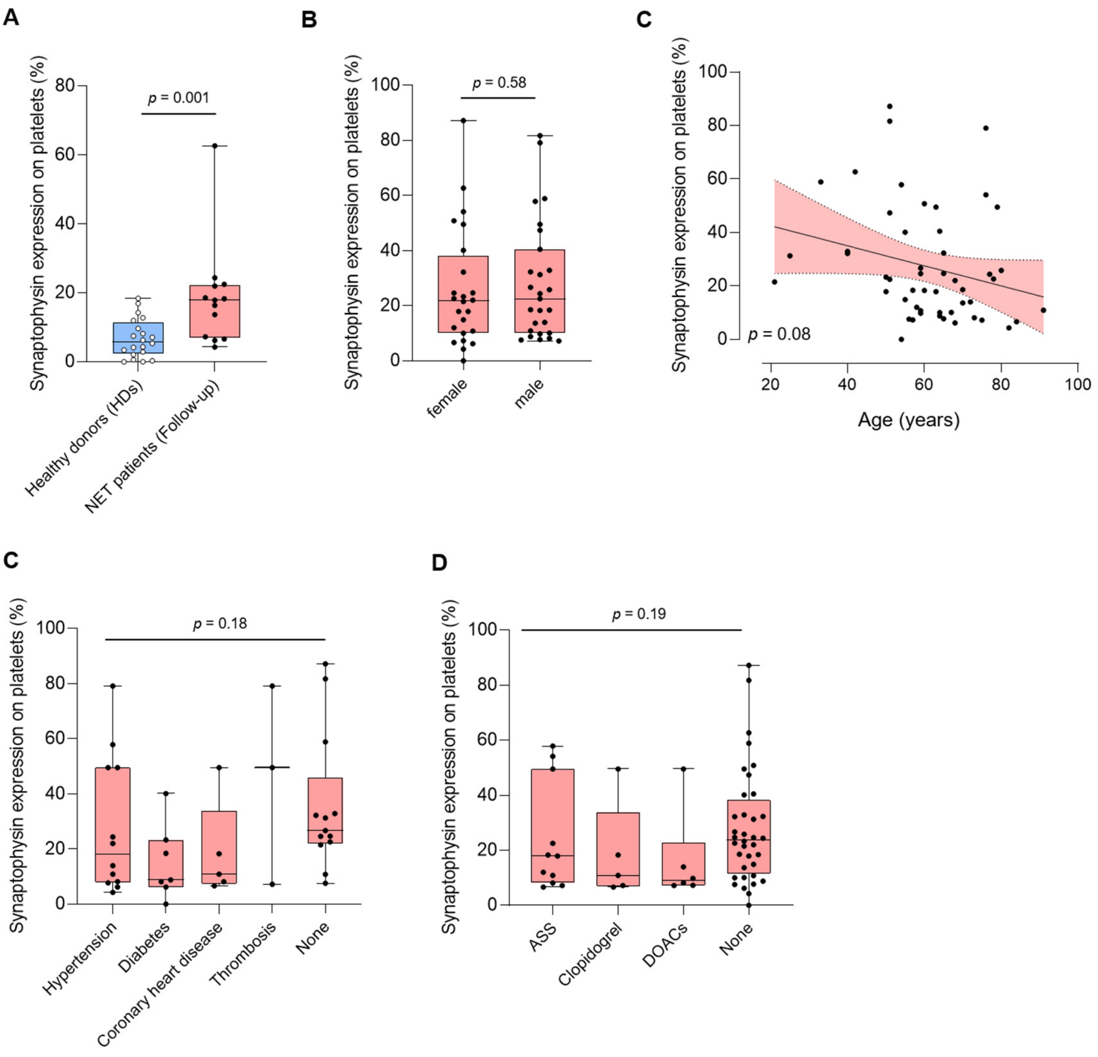

Figure S1. Correlation of pSyn expression and clinical parameters in NEN. **(A)** pSyn expression levels in healthy donors (HD) and NEN patients receiving follow-up care. **(B)** Correlation of pSyn expression and sex, age **(C)**, comorbidities **(C)** and medication affecting platelet function **(D)**.

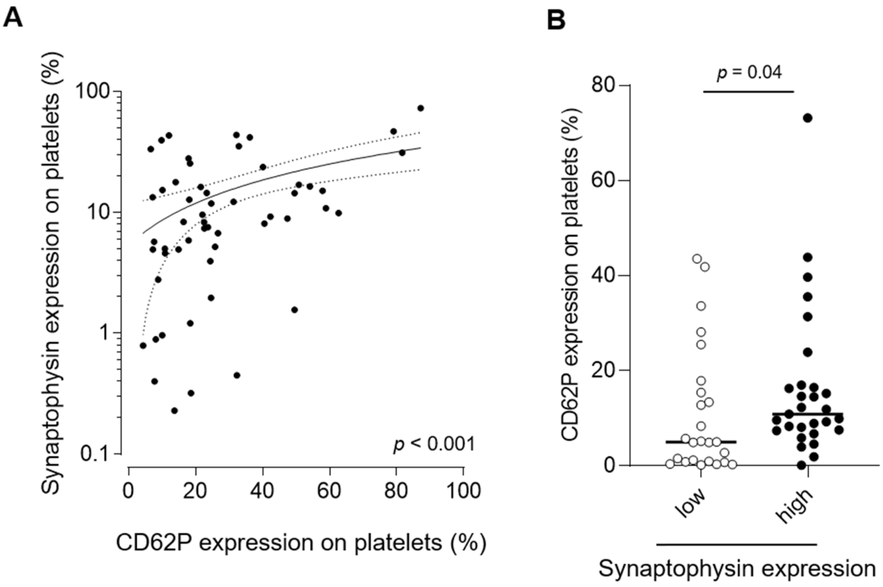

Figure S2. Association of pSyn and platelet activation. **(A)** Linear regression of pSyn expression levels and platelet activation (CD62P expression). **(B)** Platelet activation (CD62P expression) in the pSyn high (pSynhi) and pSyn low (pSynlo) cohort.

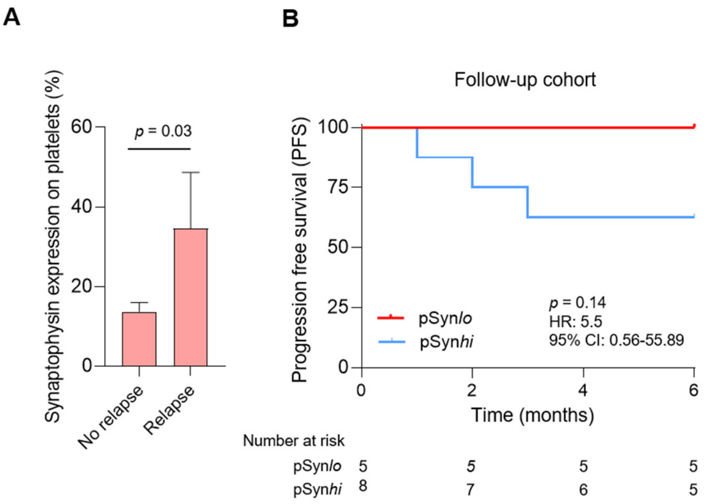

Figure S3. Expression of pSyn in NEN patients showing a disease relapse. **(A)** pSyn expression level in patients with a disease relapse (6-months follow-up after study inclusion). **(B)** Kaplan-Meier analysis for PFS in pSyn high (pSynhi) and pSyn low (pSynlo) expressing patients in the follow-up cohort 6 months after study inclusion.

Table S1. Clinical characteristics of the NEN screening cohort (SC).

**Patient Characteristics**	**Total (*n* = 54)**
Tumor origin, *n* (%)	
Ileum	19 (35.2)
Pancreas	12 (22.2)
CUP	9 (16.7)
Rectum	5 (9.3)
Duodenum	2 (3.7)
Jejunum	2 (3.7)
Lung	2 (3.7)
Stomach	2 (3.7)
Gallbladder	1 (1.9)
Treatment, *n* (%)	
Surgery, *n* (%)	39 (72.2)
Chemotherapy, *n* (%)	14 (25.9)
PRRT, *n* (%)	12 (22.2)
SIRT, *n* (%)	2 (3.7)

n = number, % = percentage, PRRT = peptide receptor radionuclide therapy, SIRT = selective internal radiation therapy.
